# Supplementary material for: Development and Characterization of Ammonia Removal Moving Bed Biofilms for Landfill Leachate Treatment
Source: Microorganisms. 2024 Nov 23;12(12):2404. doi: 10.3390/microorganisms12122404 (PMC11677484; doi:10.3390/microorganisms12122404)
Supplement: Supplementary file 1 [file microorganisms-12-02404-s001.zip › microorganisms-3240510-supplementary.pptx]

## Slide 1
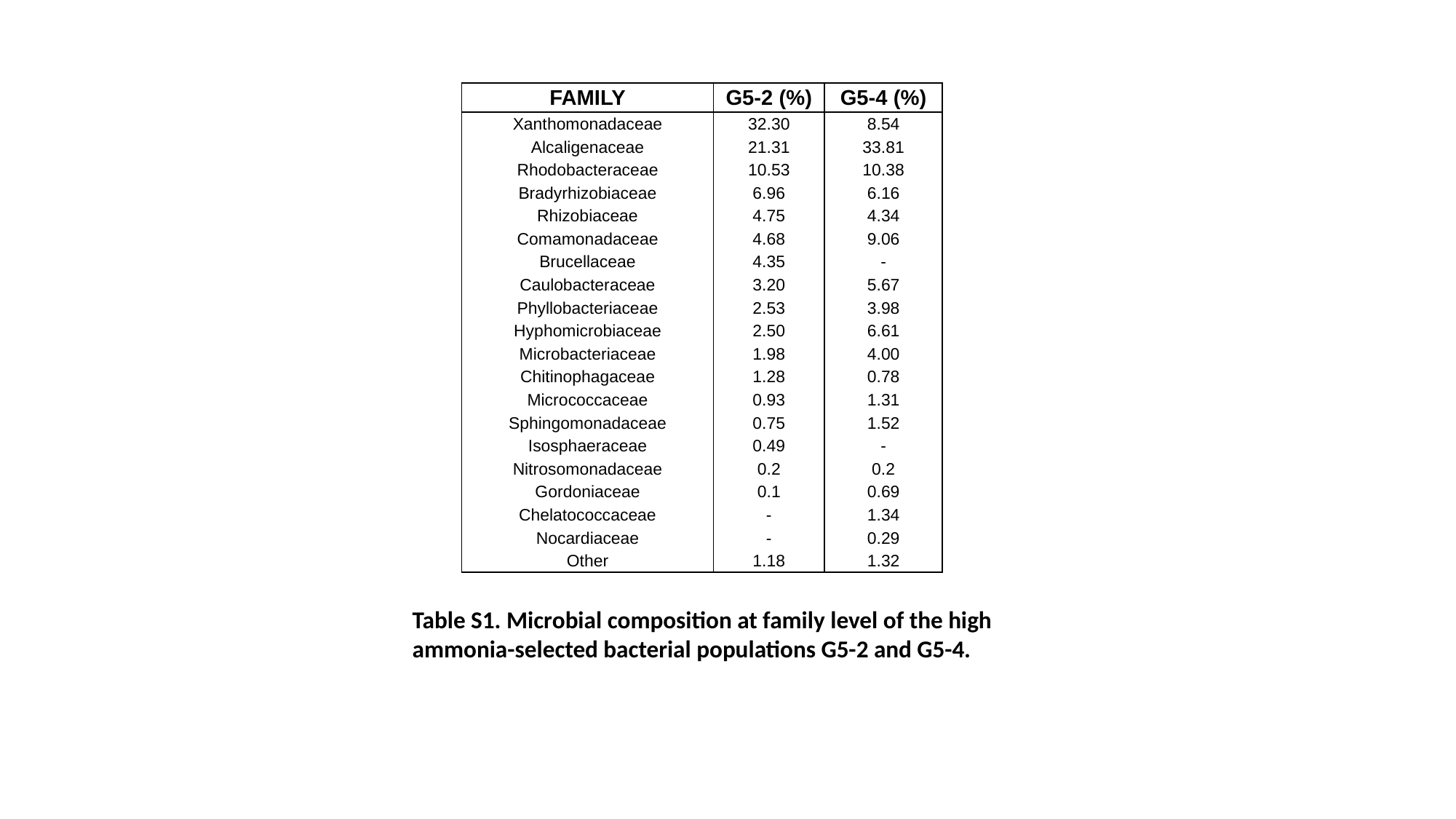

| FAMILY | G5-2 (%) | G5-4 (%) |
| --- | --- | --- |
| Xanthomonadaceae | 32.30 | 8.54 |
| Alcaligenaceae | 21.31 | 33.81 |
| Rhodobacteraceae | 10.53 | 10.38 |
| Bradyrhizobiaceae | 6.96 | 6.16 |
| Rhizobiaceae | 4.75 | 4.34 |
| Comamonadaceae | 4.68 | 9.06 |
| Brucellaceae | 4.35 | - |
| Caulobacteraceae | 3.20 | 5.67 |
| Phyllobacteriaceae | 2.53 | 3.98 |
| Hyphomicrobiaceae | 2.50 | 6.61 |
| Microbacteriaceae | 1.98 | 4.00 |
| Chitinophagaceae | 1.28 | 0.78 |
| Micrococcaceae | 0.93 | 1.31 |
| Sphingomonadaceae | 0.75 | 1.52 |
| Isosphaeraceae | 0.49 | - |
| Nitrosomonadaceae | 0.2 | 0.2 |
| Gordoniaceae | 0.1 | 0.69 |
| Chelatococcaceae | - | 1.34 |
| Nocardiaceae | - | 0.29 |
| Other | 1.18 | 1.32 |
Table S1. Microbial composition at family level of the high ammonia-selected bacterial populations G5-2 and G5-4.

## Slide 2
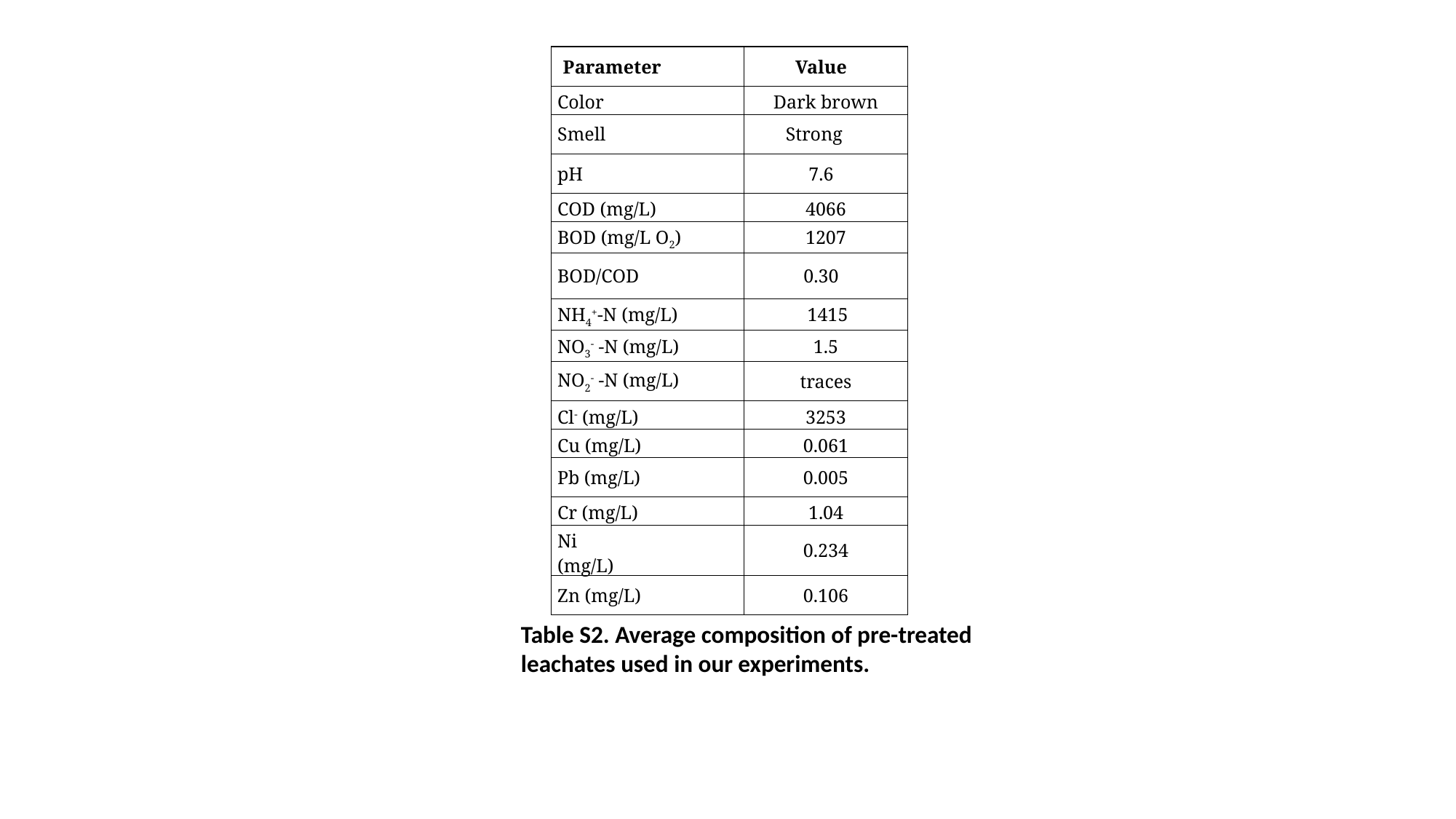

| Parameter | Value |
| --- | --- |
| Color | Dark brown |
| Smell | Strong |
| pH | 7.6 |
| COD (mg/L) | 4066 |
| BOD (mg/L O2) | 1207 |
| BOD/COD | 0.30 |
| NH4+-N (mg/L) | 1415 |
| NO3- -N (mg/L) | 1.5 |
| NO2- -N (mg/L) | traces |
| Cl- (mg/L) | 3253 |
| Cu (mg/L) | 0.061 |
| Pb (mg/L) | 0.005 |
| Cr (mg/L) | 1.04 |
| Ni (mg/L) | 0.234 |
| Zn (mg/L) | 0.106 |
Table S2. Average composition of pre-treated leachates used in our experiments.

## Slide 3
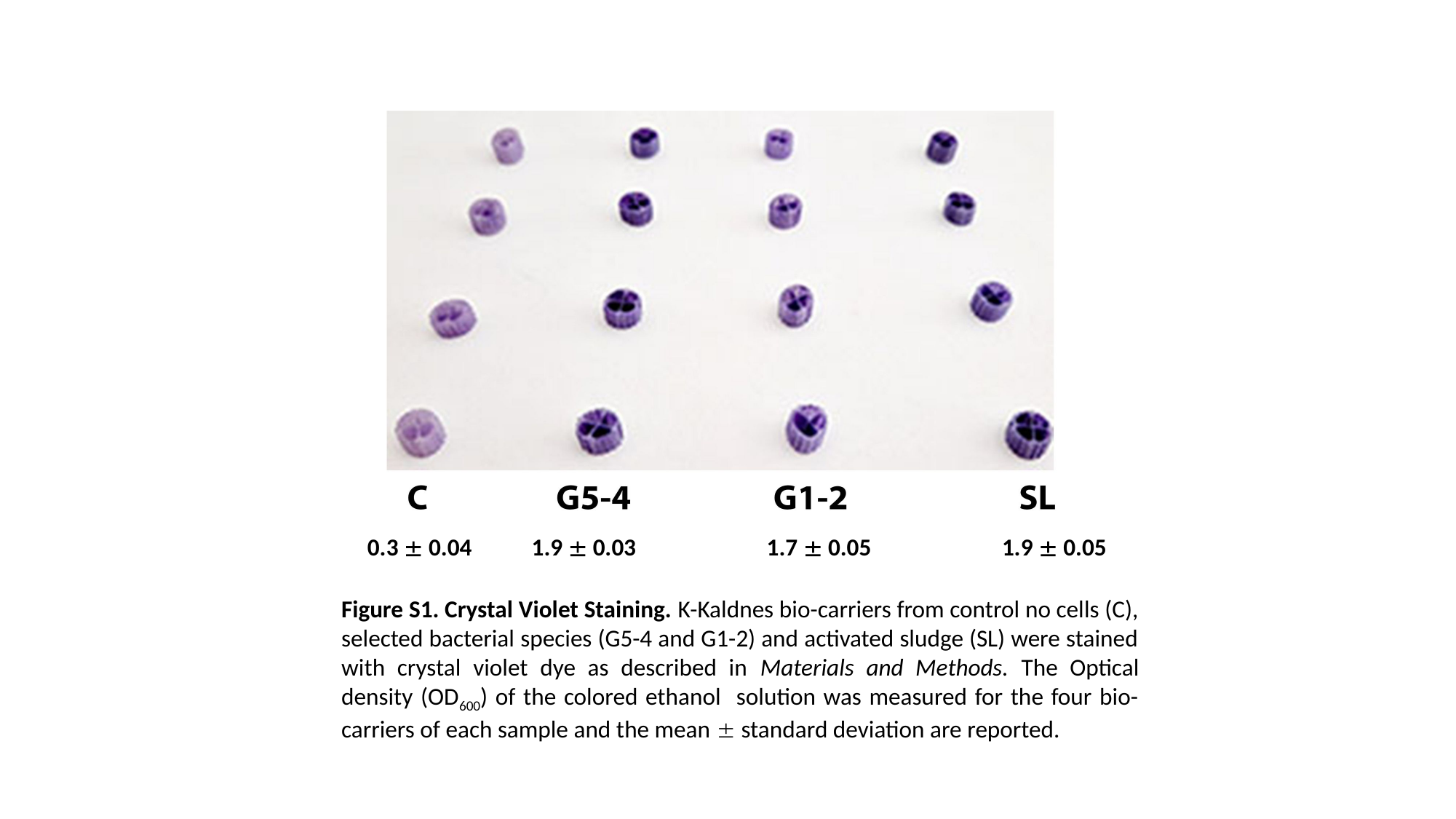

0.3 ± 0.04 1.9 ± 0.03 1.7 ± 0.05 1.9 ± 0.05
Figure S1. Crystal Violet Staining. K-Kaldnes bio-carriers from control no cells (C), selected bacterial species (G5-4 and G1-2) and activated sludge (SL) were stained with crystal violet dye as described in Materials and Methods. The Optical density (OD600) of the colored ethanol solution was measured for the four bio-carriers of each sample and the mean ± standard deviation are reported.

## Slide 4
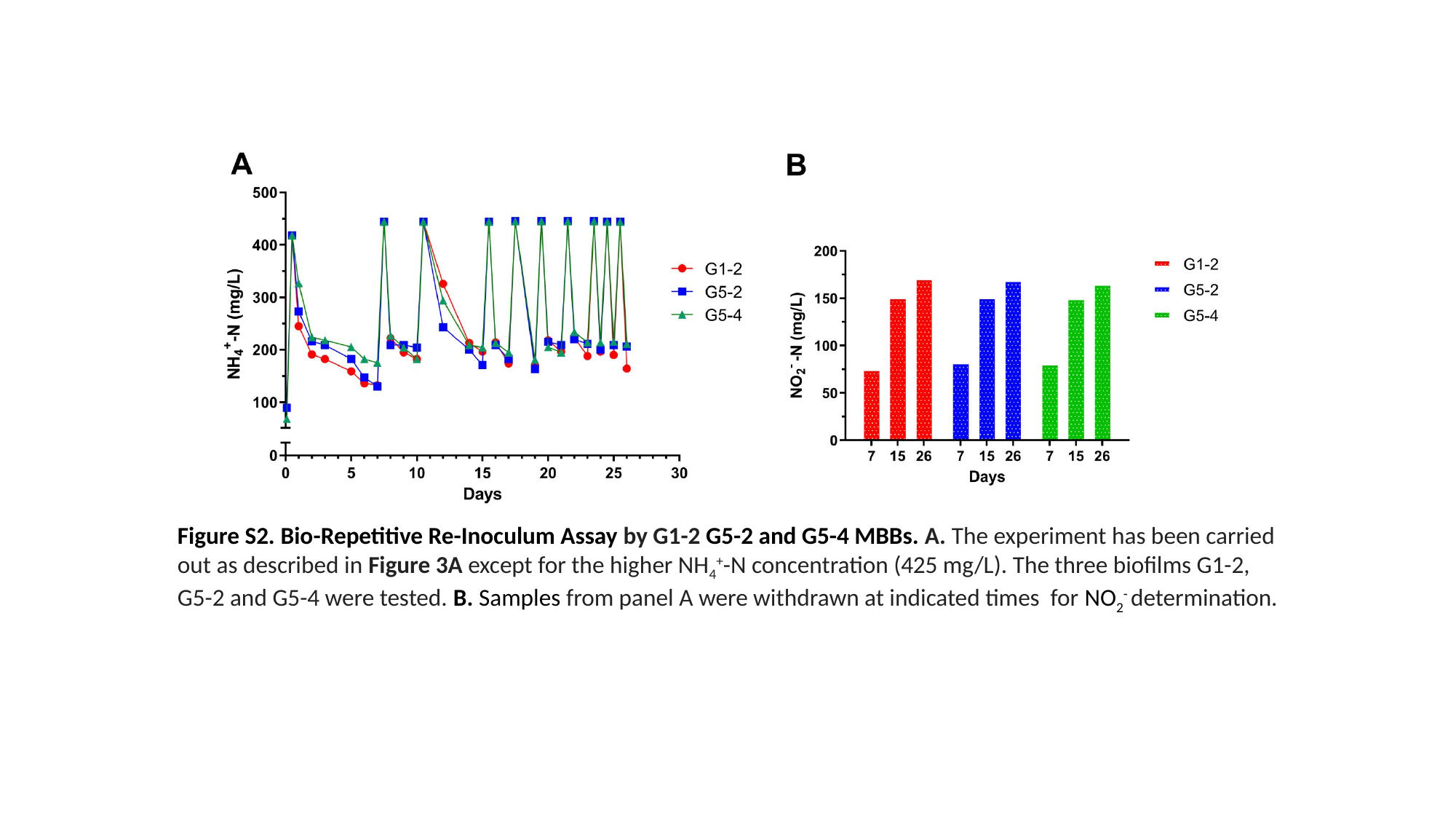

Figure S2. Bio-Repetitive Re-Inoculum Assay by G1-2 G5-2 and G5-4 MBBs. A. The experiment has been carried out as described in Figure 3A except for the higher NH4+-N concentration (425 mg/L). The three biofilms G1-2, G5-2 and G5-4 were tested. B. Samples from panel A were withdrawn at indicated times for NO2- determination.

## Slide 5
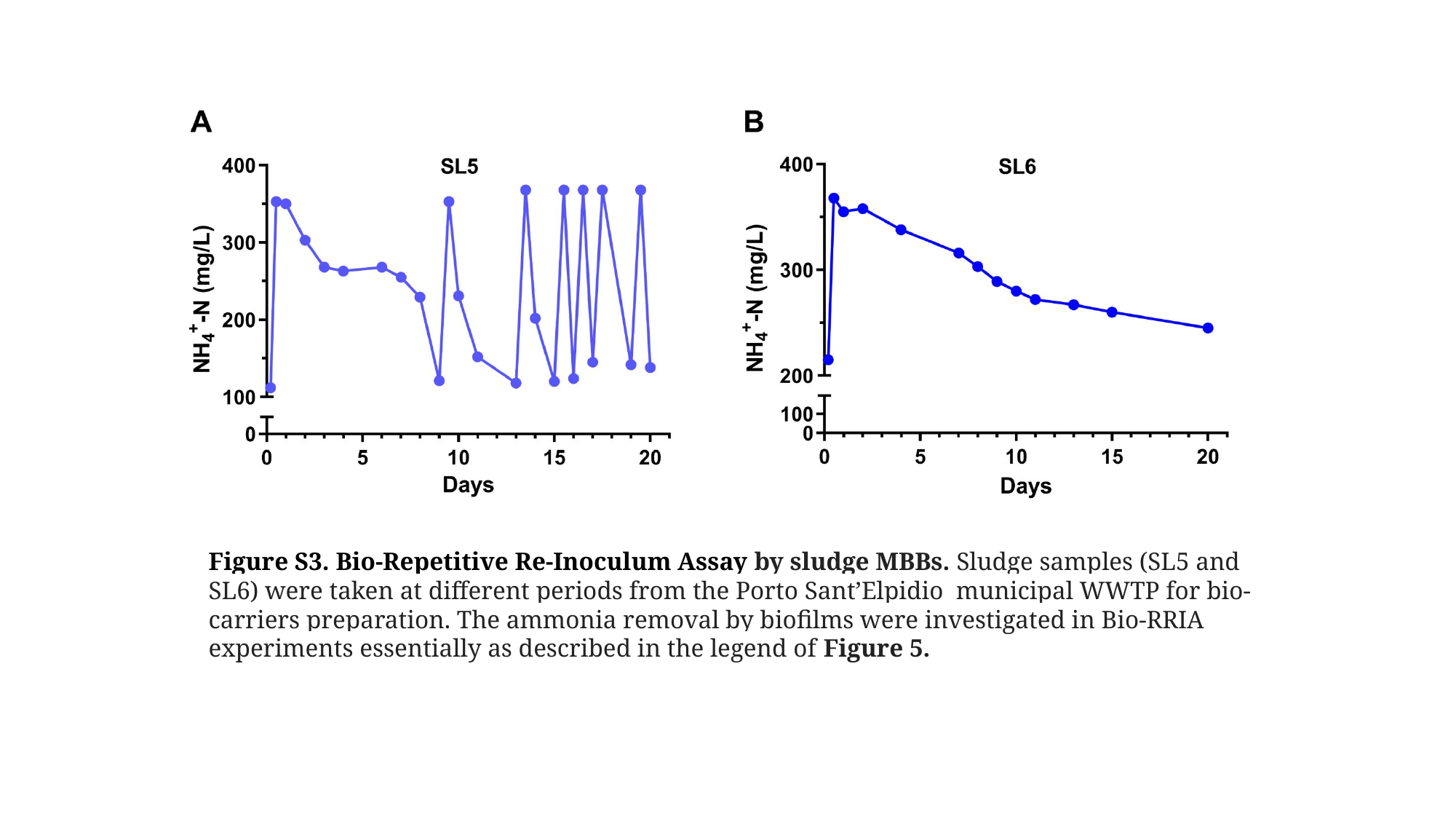

Figure S3. Bio-Repetitive Re-Inoculum Assay by sludge MBBs. Sludge samples (SL5 and SL6) were taken at different periods from the Porto Sant’Elpidio municipal WWTP for bio-carriers preparation. The ammonia removal by biofilms were investigated in Bio-RRIA experiments essentially as described in the legend of Figure 5.

## Slide 6
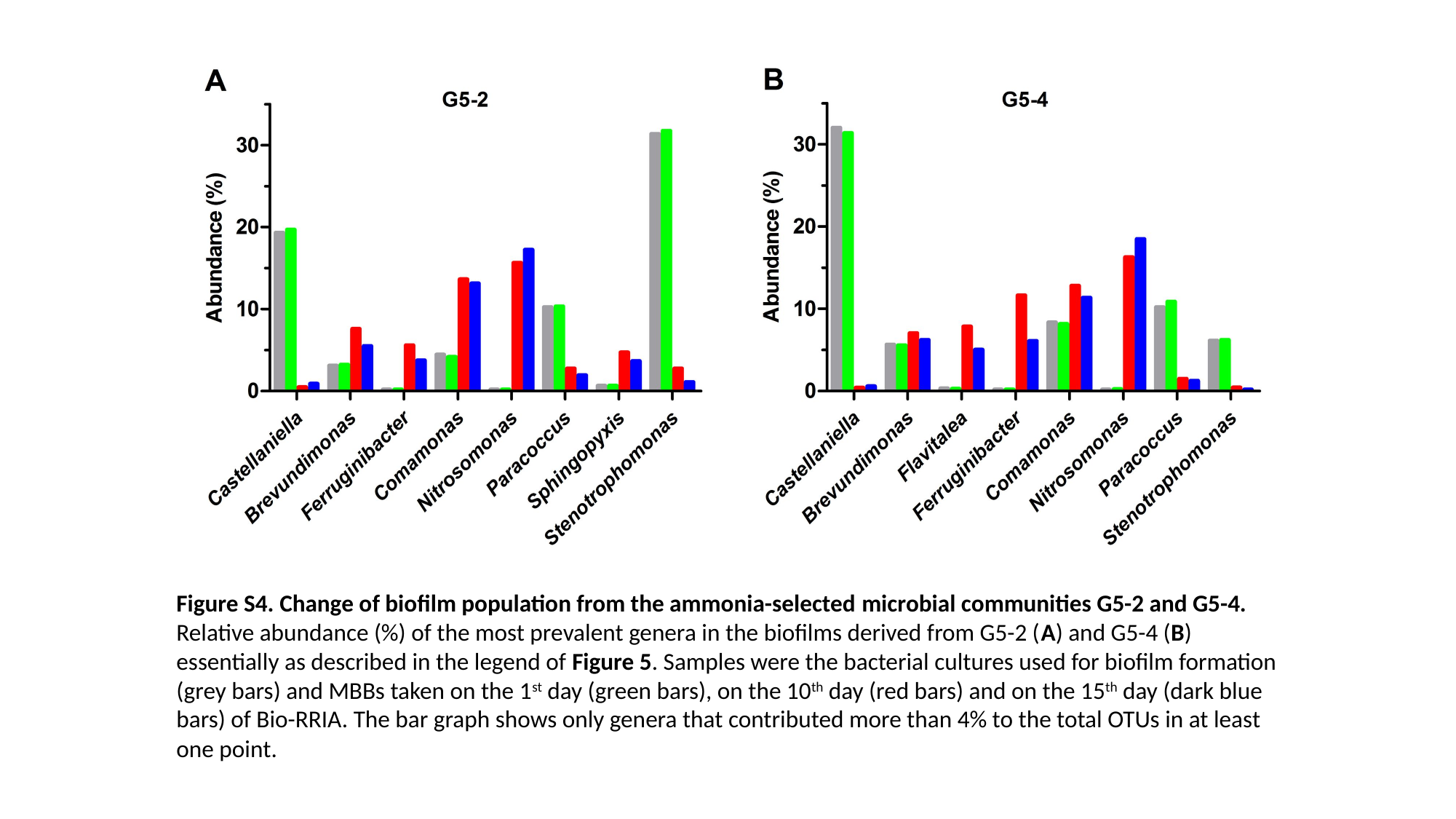

Figure S4. Change of biofilm population from the ammonia-selected microbial communities G5-2 and G5-4. Relative abundance (%) of the most prevalent genera in the biofilms derived from G5-2 (A) and G5-4 (B) essentially as described in the legend of Figure 5. Samples were the bacterial cultures used for biofilm formation (grey bars) and MBBs taken on the 1st day (green bars), on the 10th day (red bars) and on the 15th day (dark blue bars) of Bio-RRIA. The bar graph shows only genera that contributed more than 4% to the total OTUs in at least one point.
